# COVID-19 pandemic and indices of domestic and family violence against women in the city of são joão batista/sc, brazil, in the years 2019 and 2020

**DOI:** 10.1192/j.eurpsy.2023.1244

**Published:** 2023-07-19

**Authors:** P. M. D. Silva, M. E. Nunes, M. A. Cigognini

**Affiliations:** 1 Escola de Ciências Jurídicas e Sociais, Universidade do Vale do Itajaí - UNIVALI, Itajaí/SC; 2 Escola de Ciências Jurídicas e Sociais, Universidade do Vale do Itajaí - UNIVALI, Itajaí; 3 Instituto de Neurociência e Comportamente, Blumenau/SC; 4 Universidade de São Paulo - USP, São Paulo/SP, Brazil

## Abstract

**Introduction:**

According to Article 5 of the Maria da Penha Law, domestic and family violence against women constitutes any action or omission based on gender that causes her death, injury, physical, sexual or psychological suffering, and moral or patrimonial damage, within the scope of the domestic unit, the family or any intimate relationship of affection. In isolation, domestic violence is already considered by the WHO a public health problem. In the context of a pandemic, as was the case with COVID-19, the issue had the severity increase.

**Objectives:**

To investigate the rates of domestic and family violence against women in the city of São João Batista, State of Santa Catarina, Brazil, between January 1, 2019, and December 31, 2020, relating the data to the COVID-19 pandemic.

**Methods:**

Descriptive cross-sectional study, operationalized by the technique of bibliographic and documentary research.

**Results:**

There was a reduction in the total number of crimes involving domestic and family violence against women in the city of São João Batista/SC, Brazil. While in 2019 there was a notification of 116 crimes, in 2020 the number dropped to 65. (Graph 1)

The most frequent crimes in both years were: threat and bodily injury. Together they accounted for 83% of cases in 2020. (Graph 2)

The apparent decrease in the number of notifications does not necessarily represent a decrease in cases of domestic violence, but rather, it may mean fear of leaving the residence to formalize the complaint, difficulty in carrying it out, or even the worsening of factors that increase the risk in these situations, such as reduction or total loss of income and support network.

**Image:**

**Figure:**
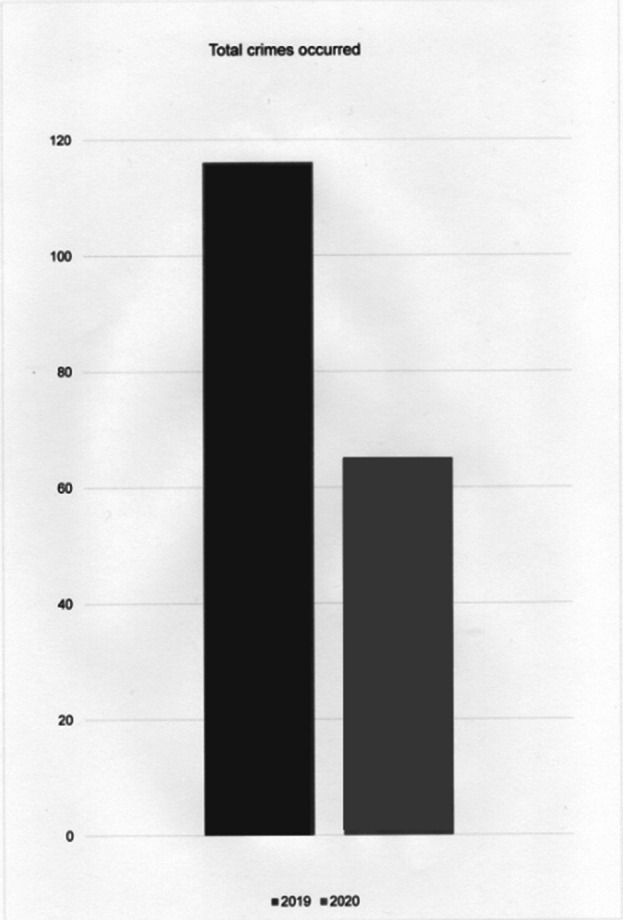

**Image 2:**

**Figure:**
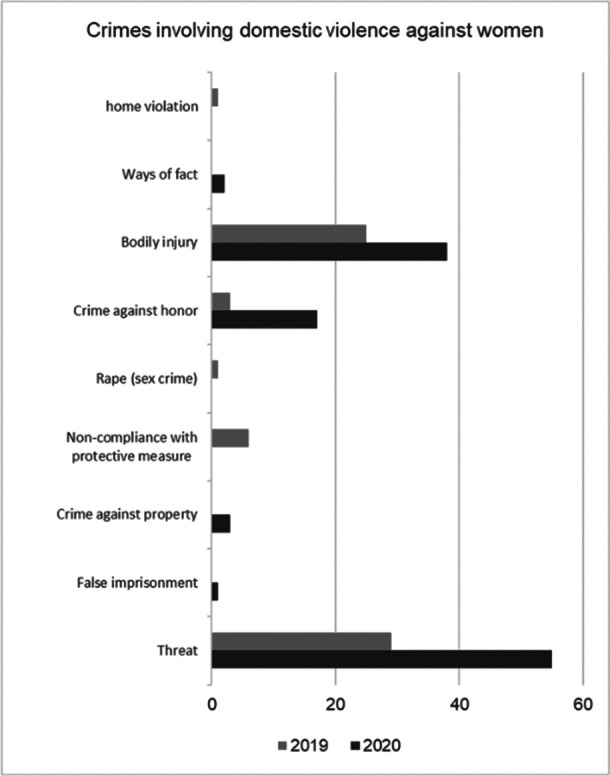

**Conclusions:**

The scenario presented shows the need for preventive public policies regarding the problem of domestic and family violence against women. The creation and strengthening of community and personal networks are fundamental.

**Disclosure of Interest:**

None Declared

